# 

**Published:** 1887-10

**Authors:** 


COLLABORATORS:

CHICAGO:

Professor E. Andrews, M.D., LL.D.
Professor J. Bartlett, M.D.
Professor W. T. Belfield, M.D.
Professor F. Billings, M.D.
Professor W. H. Byford, A.M., M.D.
Professor L. Curtis, A.M., M.D.
Professor N. S. Davis, Jr., A.M., M.D.
Professor O. C. DeWolf, A.M., M.D.
Professor E. C. Dudley, A.B., M.D.
Professor C. Fenger, M.D.
Professor J. N. Hyde, A.M., M.D.
Professor E. F. Ingals, A.M., M.D.
Professor W. W. Jaggard, A.M., M.D.
Professor F. S. Johnson, A.M., M.D.
Professor H. A. Johnson, M.D., LL.D.
Professor C. W. Purdy, M.D.
Professor C. G. Smith, A.M., M.D.
Professor E. Wing, A.M., M.D.

MILWAUKEE, WIS.
Professor N. Senn, M.D.

WASHINGTON, D. C.:

S. O. Richey, M.D.

UNITED STATES ARMY:
Surgeon D. L. Huntington, M.D.

MARINE HOSPITAL SERVICE:

Surgeon S. T. Armstrong, M.D.

UNITED STATES NAVY:
Medical Director J. M. Browne, M.D.
Medical Director A. L. Gihon, A.M., M.D.

